# The VDAC1-based R-Tf-D-LP4 Peptide as a Potential Treatment for Diabetes Mellitus

**DOI:** 10.3390/cells9020481

**Published:** 2020-02-19

**Authors:** Srinivas Pittala, Idan Levy, Soumasree De, Swaroop Kumar Pandey, Nataly Melnikov, Tehila Hyman, Varda Shoshan-Barmatz

**Affiliations:** Department of Life Sciences and the National Institute for Biotechnology in the Negev, Ben-Gurion University of the Negev, Beer-Sheva 84105, Israel; sirinivas9@gmail.com (S.P.); idan0@post.bgu.ac.il (I.L.); soumasree.de@gmail.com (S.D.); pandey@post.bgu.ac.il (S.K.P.); chepovetsky93@gmail.com (N.M.); tehilahm@post.bgu.ac.il (T.H.)

**Keywords:** β-cells, high-fat diet, mitochondria, type 2 diabetes, VDAC1-based R-Tf-D-LP4 peptide

## Abstract

Diabetes mellitus is a metabolic disorder approaching epidemic proportions. Non-alcoholic fatty liver disease (NAFLD) regularly coexists with metabolic disorders, including type 2 diabetes, obesity, and cardiovascular disease. Recently, we demonstrated that the voltage-dependent anion channel 1 (VDAC1) is involved in NAFLD. VDAC1 is an outer mitochondria membrane protein that serves as a mitochondrial gatekeeper, controlling metabolic and energy homeostasis, as well as crosstalk between the mitochondria and the rest of the cell. It is also involved in mitochondria-mediated apoptosis. Here, we demonstrate that the VDAC1-based peptide, R-Tf-D-LP4, affects several parameters of a NAFLD mouse model in which administration of streptozotocin (STZ) and high-fat diet 32 (STZ/HFD-32) led to both type 2 diabetes (T2D) and NAFLD phenotypes. We focused on diabetes, showing that R-Tf-D-LP4 peptide treatment of STZ/HFD-32 fed mice restored the elevated blood glucose back to close to normal levels, and increased the number and average size of islets and their insulin content as compared to untreated controls. Similar results were obtained when staining the islets for glucose transporter type 2. In addition, the R-Tf-D-LP4 peptide decreased the elevated glucose levels in a mouse displaying obese, diabetic, and metabolic symptoms due to a mutation in the obese (ob) gene. To explore the cause of the peptide-induced improvement in the endocrine pancreas phenotype, we analyzed the expression levels of the proliferation marker, Ki-67, and found it to be increased in the islets of STZ/HFD-32 fed mice treated with the R-Tf-D-LP4 peptide. Moreover, peptide treatment of STZ/HFD-32 fed mice caused an increase in the expression of β-cell maturation and differentiation PDX1 transcription factor that enhances the expression of the insulin-encoding gene, and is essential for islet development, function, proliferation, and maintenance of glucose homeostasis in the pancreas. This increase occurred mainly in the β-cells, suggesting that the source of their increased number after R-Tf-D-LP4 peptide treatment was most likely due to β-cell proliferation. These results suggest that the VDAC1-based R-Tf-D-LP4 peptide has potential as a treatment for diabetes.

## 1. Introduction

The voltage-dependent anion channel 1 (VDAC1) is a multi-functional protein located in the outer mitochondrial membrane (OMM), and is a key regulator of mitochondrial function [1,2,3,4,5]. VDAC1 has been shown to serve as a mitochondrial gatekeeper, controlling the metabolic and energetic crosstalk between mitochondria and the rest of the cell, and it is also one of the key proteins in mitochondria-mediated apoptosis [1,2,3,5]. Its importance in cell energy and metabolism homeostasis is reflected by the findings that downregulation of VDAC expression decreased metabolite exchange between mitochondria and the cytosol, and inhibited cell growth [1,2,3]. Under physiological conditions, VDAC1 is present as both a monomer and dimer; however, upon induction of apoptosis, the VDAC1 dimers undergo conformational changes to assemble into higher oligomeric states [6,7,8,9]. VDAC1 oligomerization has been proposed to play an important role in apoptosis by mediating cytochrome c (Cyto c) release, and regulating apoptosis by binding apoptosis-regulating proteins [7,8,10]. 

VDAC1, involved in many cellular processes, including metabolism, Ca^2+^ homeostasis, apoptosis, and other activities, is regulated via its interaction with the relevant proteins associated with these activities [3,9,11,12]. In fact, VDAC1 appears to be a convergence point for a variety of cell survival and death signals that are mediated via association with ligands and proteins, and it is considered a hub protein that interacts with over 100 others. The VDAC1 protein–protein interaction (PPI) networks include proteins involved in metabolism, apoptosis, and the cytoskeleton, in addition to signaling-related proteins that regulate the integration of mitochondrial functions with other cellular activities [13,14,15]. 

Given the variety of roles played by VDAC1 in cell metabolism and apoptosis and its interaction with many partners [4,5,16,17,18,19,20], modulating such interactions by VDAC1-based peptides represents a potential therapeutic modality for treating metabolic disorders such as non-alcoholic fatty liver disease (NAFLD) and type 2 diabetes (T2D). Accordingly, we have designed VDAC1-based peptides targeting these interactions [17,18,20,21,22]. One of these peptides—R-Tf-D-LP4—was selected for this study.

The R-Tf-D-LP4 peptide comprises a VDAC1-derived sequence defined as LP4, fused to a cell-penetrating peptide—the human transferrin receptor (hTfR)-recognition sequence HAIYPRH (Tf) [22]. The amino acids of the VDAC1-derived sequence are in the D-configuration [18], promoting higher oral bioavailability, and are less immunogenic than the corresponding L-peptides [22]. In the retro-inverso R-Tf-D-LP4 peptide, the direction of the R-Tf-D-LP4 peptide bond is reversed, producing a side chain topology similar to that of the original L-amino acid R-Tf-D-LP4 peptide [22]. 

This R-Tf-D-LP4 peptide was found to normalize blood glucose in the streptozotocin (N-acetyl-β-D-glucosaminidase inhibitor; STZ) and high-fat diet (HFD-32)-fed mouse model (STZ/HFD-32), while it reversed liver pathology to a normal-like state [23]. 

In the STZ/HFD-32 mouse model used, STZ is administered neonatally to produce mild systemic inflammation and hyperglycemia, which is then followed by a high-fat diet challenge starting at postnatal week four. The dose of STZ is designed to produce a partial impairment of insulin secretion, and the resultant phenotype is similar to advanced T2D where beta cells are functionally exhausted and fail to secrete insulin at normal levels [24].

Treatment of STZ/HFD-32 mice with R-Tf-D-LP4 peptide affected carbohydrate and lipid metabolism, and increased the expression of enzymes and factors associated with fatty acid transport to mitochondria, enhancing β-oxidation and thermogenic processes, while decreasing the expression of enzymes and regulators of fatty acid synthesis [23]. The STZ/HFD-32 mouse model exhibited increased blood glucose levels, which were reduced by treatment with the R-Tf-D-LP4 peptide to the levels normally found in chow diet-fed mice [23]. 

It is now generally accepted that the presence of NAFLD and progressive non-alcoholic steatohepatitis (NASH) are associated with an increased incidence of T2D [25]. Often, NAFLD and T2D coexist and act synergistically to drive adverse clinical outcomes, where the presence of NAFLD increases the incidence of T2D and accelerates the development of diabetic complications [26,27]. NAFLD affects 20 to 30 percent of the general population in various countries, is more prevalent in males than females [28], and is often associated with insulin resistance, obesity, and diabetes mellitus (DM). 

DM is a chronic metabolic disorder characterized by hyperglycemia [29], with about 400 million individuals reported to suffer from T2D worldwide, and approximately 320 million diagnosed with prediabetes (http://www.idf.org/action-on-diabetes/sdgs). It is a result of insulin dysfunction, either due to defects in insulin secretion, insulin action, or both [30]. Insulin acts as the main anabolic hormone by binding the insulin receptor on target cells and propagating an intra-cellular response, mainly the fusion of vesicles containing the glucose transporter 4 (Glut 4) to the plasma membrane, thereby enhancing cell glucose uptake [31]. Type 1 diabetes (T1D) is characterized by insufficient insulin secretion due to complete destruction of the insulin-producing β-cells of the pancreas [32,33], while T2D more commonly results from insulin resistance, namely a reduced response to insulin by the target tissues [30]. The β-cells respond to insulin resistance by increased insulin production although, at some point, the cells become exhausted with resulting hyperglycemia when the production of insulin does not match the increased insulin demand. 

As already discussed, there is a high degree of association between T2D and NAFLD. A dysfunctional liver, such as seen in NAFLD, may dysregulate insulin action [34,35]. The abnormal glucose metabolism is a common phenotype observed in NASH, and there appears to be a strong link between NASH and T2D, with 44% of NASH patients estimated to have diabetes [36]. Mitochondrial dysfunction has been implicated in both NAFLD and diabetes, as well as in insulin resistance, which is shared by both conditions [37,38,39]. Dysfunctional mitochondria may further promote diabetes and NAFLD by impairing the energy homeostasis in hepatocytes or insulin target cells, thereby inducing an abnormal accumulation of lipids in hepatocytes or a reduced response to insulin [40,41].

Our previous studies demonstrated that VDAC1 is upregulated and participates in diabetes-related dysfunction in the leptin-deficient db/db mouse model of T2D [42]. We showed that, in these mice, VDAC1 is mistargeted to the plasma membrane of insulin-secreting β-cells with a loss of cellular ATP and a consequent inhibition of depolarization-induced insulin secretion [42]. Treatment of db/db mice with a VDAC1 inhibitor, VBIT-4 [43], prevented hyperglycemia, stimulated insulin secretion, and maintained normal glucose tolerance [42]. 

The current study was designed to investigate the effect of the R-Tf-D-LP4 peptide on blood glucose in STZ/HFD-32 fed and ob/ob mice, while focusing on the pancreas. Treatment with R-Tf-D-LP4 reduces blood glucose levels, and increases the number of insulin-producing β-cells in islets, in addition to increasing the number and size of the islets and improving their morphology. We attribute the increase in β-cells to an induction of proliferation by this peptide. 

## 2. Materials and Methods

### 2.1. Materials

Citrate, 4′,6-diamidino-2-phenylindole (DAPI), Tris, trisodium, streptozotocin, Triton X-100, and Tween-20 were purchased from Sigma (St. Louis, MO). Eosin, hematoxylin, and Oil Red O were purchased from Fisher Scientific (Geel, Belgium). Dimethyl sulfoxide (DMSO) was purchased from MP Biomedicals (Solon, OH). Formaldehyde was purchased from Emsdiasum (Hatfield, PA). 3,3-diaminobenzidine (DAB) was obtained from ImmPact-DAB (Burlingame, CA), and a Performa Accu-Chek glucometer and test strips from Roche (Indianapolis, IN). Dulbecco’s modified Eagle’s medium (DMEM), was purchased from Gibco (Grand Island, NY). The sources and dilutions of primary and secondary antibodies are detailed in Table 1. R-Tf-D-LP4 R-Tf-D-LP4 peptide (KWTWK-216-NSNGATWALNVATELKK-199-EWTWSHRPYIAH), comprising 34 residues in D-configuration (except for the underlined Tf sequence) was synthesized by GL Biochem (Shanghai, China) to > 95% purity. The R-Tf-D-LP4 peptide was first dissolved in DMSO as a 40-mM solution and then diluted 20-fold in the appropriate buffer. The R-Tf-D-LP4 peptide concentration was determined using absorbance at 280 nm and the specific molar excitation coefficient.

### 2.2. Dietary Intervention

The HFD-32 diet, as described previously [44] (507.6 kcal/100 g, 56.7% kcal from fat), comprised 5% egg white powder (MM Ingredients, Wimborne, UK), 6.928% lactose (Pharma Grade, Nelson, UK), 15.88% beef fat (contains 80% fat), 5% AIN93G-mineral mixture, 0.002% tertiary butyl hydroquinone and 1.4% AIN93VX-vitamin mix (MP Biomedical, Illkirch, France), 24.5% milk casein (Shaanxi Fuheng Biotechnology, Xi’an, China), 20% safflower oil (high oleic acid content) (Bustan Briut, Galil, Israel), 6.45% sucrose, 0.43% L-cysteine, 5.5% crystalline cellulose (Sigma), 0.36% choline bitartrate, and 8.25% maltodextrin (Bulk Powders, Colchester, UK). Control C57BL/6 mice were fed with a standard chow diet (408.4 kcal/100g, 57% kcal from carbohydrates, 27% kcal from proteins, 16% from fat (V1154-703, Ssniff Spezialdäten, Sosset, Germany).

### 2.3. NAFLD/diabetes Mouse Model 

Male C57BL/6 mice were purchased from Envigo (Jerusalem, Israel). In order to generate the diabetes-based steatosis-NASH mouse model, 2-day-old mice were subjected to a single low-dose subcutaneous injection of streptozotocin (STZ) (200 µg/mouse) and were then fed the HFD-32 high-fat diet from the end of Week 4. The mice manifested steatosis at 6 weeks and NASH at 8 weeks. They were treated with R-Tf-D-LP4 (14 mg/kg) from Week 6 to Week 8 for steatosis, or from Week 8 to Week 10 for NASH. The R-Tf-D-LP4 peptide at 14 mg/kg [in 100 µL of Hank’s balanced salt solution (HBSS) without calcium] was intravenously administered three times a week. Control groups were intravenously injected with 100 μL of 0.8% DMSO in HBSS buffer from Week 6 to Week 8 for steatosis and from Week 8 to Week 12 for NASH. The final blood DMSO concentration was 0.07% in both control and peptide-treated mice.

At the end of the experiment, the mice were sacrificed by CO_2_ inhalation, and the liver and pancreas was removed. Livers were photographed, and the lipid content was assessed. For this purpose, part of the liver was frozen in OCT (optimal cutting temperature) compound, embedded, sectioned, and stained with Oil Red O. The pancreases were fixed with formaldehyde, embedded in paraffin, sectioned, and subjected to immunohistochemical (IHC) or immunofluorescent (IF) staining, as described below.

The experimental protocol used in the mouse model was approved by the Institutional Animal Care and Use Committee.

### 2.4. Ob/Ob Diabetes Mouse Model 

Six-week-old male (C57BL/6) ob/ob (Jackson Laboratory, Bar Harbor, ME) mice were treated from Week 6 to Week 13 with R-Tf-D-LP4 (14 mg/Kg) by intravenous administration of the R-Tf-D-LP4 peptide in 100 µL of Hank’s balanced salt solution (HBSS) without calcium three times a week. A control group was intravenously injected with 100 μL of 0.8% DMSO in HBSS buffer. The final blood DMSO concentration was 0.07% in both control and peptide-treated mice. Blood glucose levels were measured once a week. At the end of the experiment, the mice were sacrificed, and the pancreas was removed and fixed with formaldehyde, embedded in paraffin, sectioned, and subjected to IF. The experimental protocol used in the mouse model was approved by the Institutional Animal Care and Use Committee.

### 2.5. Cell Culture and R-Tf-D-LP4 Peptide Treatment

3T3-F442A (00070654) adipocyte cell line were grown and maintained in a humidified atmosphere of 5% CO_2_, 95% air at 37 °C in Dulbecco’s modified Eagle’s growth medium (DMEM) with glucose (4.5 mM), supplemented with 10% fetal calf serum, 1% penicillin-streptomycin and 5μg/mL insulin. Cells were differentiated into adipocytes once confluent. For R-Tf-D-LP4 peptide treatment, 3T3-F442A adipocytes (6 × 10^5^/mL at ~80% confluence) were incubated with the R-Tf-D-LP4 peptide in 500 µL medium for 24 h at 37 °C and 5% CO_2_. The cells were then stained with Oil Red O as described below.

### 2.6. Oil Red O Staining

Following sacrifice, the mouse livers were removed, and isolated livers were immediately embedded in OCT medium and kept at −80 °C until sectioning. Sections (10-μm thick) were washed with PBS, fixed with 4% formaldehyde for 10 min, gently washed with 60% isopropanol, and then stained with a solution of 0.5 g Oil Red O in 60% isopropanol for 15 min. The stained sections were washed several times with distilled water to remove unbound dye. The samples were then counter-stained with hematoxylin for 5 min, and images were collected using a light microscope (Leica DM2500) with the same light intensity and exposure time.

3T3-L1 cells were washed with PBS, fixed with formaldehyde for 15 min, and then subjected to Oil Red O staining for 30 min at room temperature, followed by staining with hematoxylin. Microscope images were collected to visualize the red oil droplets in the cells. Finally, the cells were washed with H_2_O followed by three washes with 60% isopropanol with gentle rocking. The Oil Red O stain was extracted with 100% isopropanol for 5 min, and the absorbance at 492 nm was measured and normalized to the same number of cells counted before stain extraction.

### 2.7. Immunohistochemistry and Immunofluorescence Analysis of Pancreas Tissue

Immunohistochemistry (IHC) and *immunofluorescence* (IF) staining were performed on 5-µm thick formalin-fixed and paraffin-embedded pancreatic tissue sections. Sections were deparaffinized (5 min in xylene, 3 times), followed by rehydration with a graded ethanol series (50, 70, 95, 100 %). Antigen retrieval was performed by 20-min incubation in preheated 0.01 M citrate buffer, pH 6.0 at 95–98 °C. Sections were washed with PBS pH 7.4 containing 0.1% Triton-X100 (PBST), incubated in 10% NGS and 1% BSA for 2 h, followed by overnight incubation at 4 °C with primary antibodies (see Table 1). Sections were then washed with PBST. For IHC staining, endogenous peroxidase activity was blocked by incubating the sections in 3% H_2_O_2_ for 15 min. Following washing with PBST, sections were incubated for 2 h with the appropriate secondary HRP-conjugated antibodies. Sections were washed with PBST, and peroxidase activity was visualized by incubating with DAB. After rinsing in water, the sections were counter-stained with hematoxylin, dehydrated with a graded ethanol series (50–100%), incubated in xylene, and mounted with mounting medium. Finally, images from the sections were collected using an automatic digital slide scanner (Panoramic MIDI II, 3DHISTECH) with the same light intensity and exposure time. Non-specific control experiments were carried out using the same protocols, but omitting incubation with primary antibodies. For IF staining, sections were incubated for 2 h with the appropriate secondary Alexa Fluor-conjugated antibodies. Sections were washed with PBST, counter-stained with DAPI (0.07 μg/mL), washed with PBST, and mounted with mounting media. The slides were then viewed with an Olympus IX81 confocal microscope or scanned using an automatic digital slide scanner.

### 2.8. Blood Glucose Measurement

Blood was collected in a capillary tube by retro orbital bleeding as described previously [45] from the STZ/HFD-32 fed mice at the end of study before sacrifice. In the case of the ob/ob mice, weekly blood samples were collected from the tail, and blood glucose levels were measured immediately using an Accu-Check Performa blood glucose meter.

### 2.9. Statistics and Data Analysis

The mean ± SEM of results obtained from at least two independent experiments with the data derived from several mice in each experiment (n = 5–8 for each group) are presented. One-way analysis of variance (ANOVA) followed by a Mann–Whitney post hoc test using Graphpad Prism 6 was employed to evaluate significant differences between the experimental groups. P-values were: * *p* < 0.05, ** *p* < 0.01, *** *p* < 0.001, and **** *p* < 0.0001.

## 3. Results

**Figure 1:**
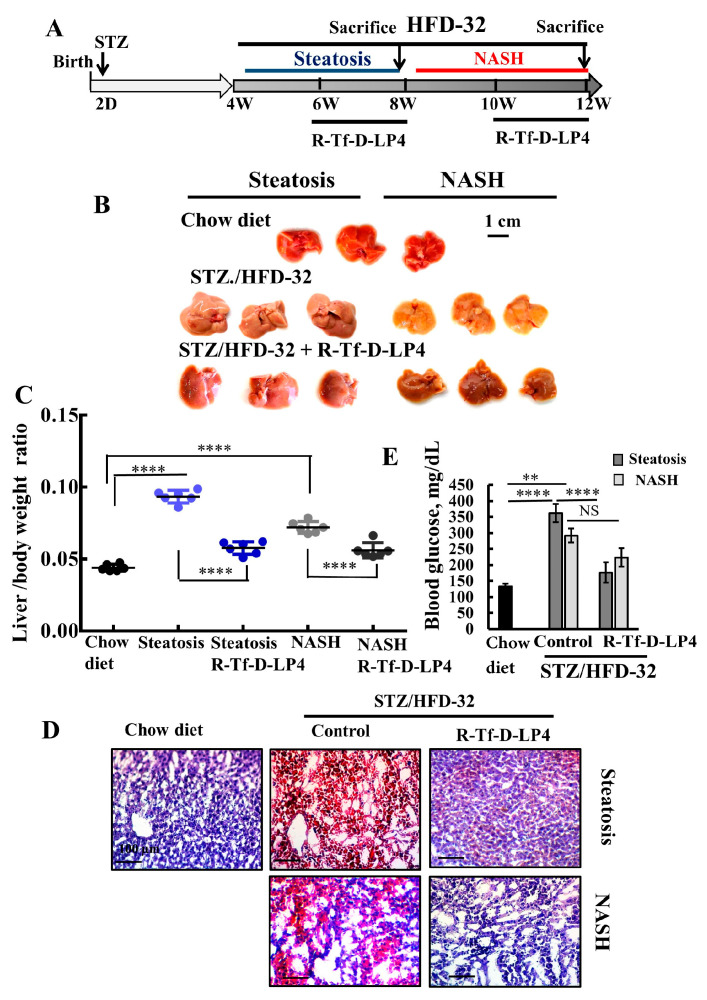
R-Tf-D-LP4 peptide-mediated inhibition of steatotic and non-alcoholic steatohepatitis (NASH) liver pathology in a STZ/HFD-32 mouse model. (**A**). Schematic presentation of the course of steatosis and NASH induced by a STZ/HFD-32 diet and the effect of R-Tf-D-LP4 peptide treatment. (**B**–**D**). Liver from mice fed with chow (normal diet), HFD-32, or HFD-32 and treated with the R-Tf-D-LP4 peptide (14 mg/kg) by i.v. injection every two days from Week 6 to 8 for steatosis and from Week 8 to 10 for NASH, as described in the Methods section. Mice were then sacrificed, livers were removed, photographed **(B),** and weighed (**C**) Results are means ± SEM (n = 10), (*p* **** ≤ 0.0001). Representative liver sections were stained with Oil Red O (**D**). Blood glucose level of mice was measured. Results are means ± SEM (n = 5–10; ** *p* ≤ 0.01, *p* **** ≤ 0.0001) (**E**).

In this study, we examined the effects of the VDAC1-derived peptide, R-Tf-D-LP4, on several parameters of a NAFLD model on a diabetic background (STZ/HFD-32) [44], with the focus on diabetes. Two-day-old mice were injected with streptozotocin (STZ), and then from 4-weeks old were fed a high-fat diet (HFD-32) (Figure 1A). Mice were treated with either DMSO 0.8% (control group) or with the R-Tf-D-LP4 peptide 14 mg/kg (Figure 1A) starting from week six after birth for steatosis, or at week eight for NASH. Mice fed a chow diet served as controls.

### 3.1. R-Tf-D-LP4 Eliminates Steatosis and NASH Pathology

To demonstrate the induction of T2D, steatosis, and NASH and the effects of the VDAC1-based peptide, R-Tf-D-LP4, we first analyzed the livers for several characteristic parameters of these pathological states (Figure 1). Livers obtained from HFD-32 fed mice had a yellow color, indicating fat accumulation, and reverted to a color similar to that of the chow diet-fed mice in the group treated with the R-Tf-D-LP4 peptide (Figure 1B). The increase in the weight of the livers in the HFD-32 fed mice with steatosis or NASH states also returned to close to normal in the R-Tf-D-LP4 peptide-treated mice (Figure 1C).

When the fat content in the livers was analyzed by Oil Red O staining of formaldehyde-fixed liver sections, the result showed high levels of staining of fat droplets in liver cells from mice suffering from either steatosis or NASH. These levels were reduced to the levels in chow diet-fed mice after treatment with the R-Tf-D-LP4 peptide (Figure 1D).

Blood glucose levels measured before sacrifice showed higher levels in the STZ/HFD-32 diet-fed mice than chow diet-fed mice, and again the levels were normalized and significantly reduced in the R-Tf-D-LP4 peptide-treated group (Figure 1E).

These results indicate that treatment with the R-Tf-D-LP4 peptide led to a reduction in NAFLD symptoms and reverted the diabetic phenotype of the model mice to close to that of the chow diet-fed mice. In order to investigate possible causes for the R-Tf-D-LP4 peptide-induced decrease in the blood glucose levels of the STZ/HFD-32 diet-fed mice, pancreases were harvested and subjected to immunohistochemical (IHC) or immunofluorescent (IF) staining for insulin, glucagon, and other proteins as presented below. 

### 3.2. Effects of R-Tf-D-LP4 Peptide of Blood Glucose Level in ob/ob Mice

The ob/ob leptin-deficient mouse is a commonly used murine model for diabetes and obesity. These mice are hyperglycemic, hyperinsulinemic, hyperlipidemic, and insulin resistant [46,47], which makes them suitable for evaluating the effects of various factors on obesity and hyperglycemia.

**Figure 2:**
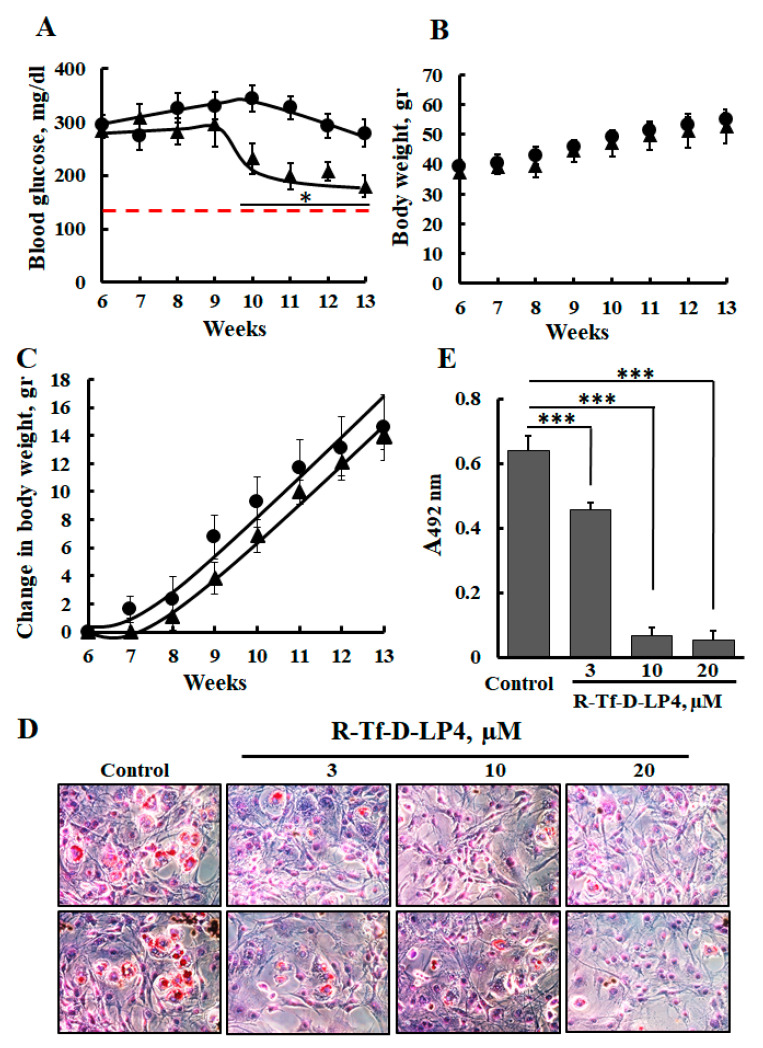
R-Tf-D-LP4 peptide treatment reduces the levels of blood glucose in ob/ob mice and decreases fat accumulation in 3T3-F442A adipocytes. (**A**) ob/ob mice were untreated (●) or treated (▲) with intravenous injections of the R-Tf-D-LP4 peptide (14 mg/kg) three times a week, and blood was drawn from an incision in the tail to measure glucose levels. The data represent the means ± SD (n = 6 mice). *p < 0.05. The dashed red line represents the blood glucose levels of WT mice. (**B**) Body weight of ob/ob mice treated with the R-Tf-D-LP4 peptide (14 mg/kg) as followed for 7 weeks. (**C**) The change in body weight as a function of time is presented. (**D**) 3T3- F442A cells were incubated with the indicated concentration of the R-Tf-D-LP4 peptide for 24 h and then stained with Oil Red O and photographed to visualize the lipid droplets stained in red. (**E**) Quantification of Oil Red O stain was carried out by stain extraction with 100% isopropanol for 5 min and measurement of the absorbance at 492 nm. The results are means ± SEM (n = 3; *** *p* ≤ 0.001).

In STZ/HFD-32 fed mice, the R-Tf-D-LP4 peptide accelerated β-oxidation in the liver [23]. Thus, we expected the R-Tf-D-LP4 peptide to affect the weight of the ob/ob mice. However, no such effect was obtained, and the weekly average weight was found to be similar whether or not the ob/ob mice were treated with the R-Tf-D-LP4 peptide (Figure 2B). Since it was possible that the peptide could not reach the fat tissue, we used 3T3-F442A adipocytes to test the effect of the R-Tf-D-LP4 peptide on the storage of intracellular lipids as visualized by Oil Red O staining (Figure 2C). The results showed that treatment with 10 μM R-Tf-D-LP4 peptide decreased the amount of lipid in 3T3-F442A adipocytes by 90% as quantified by extraction of Oil Red O stain (Figure 2D). Therefore, it is likely that the R-Tf-D-LP4 peptide either accelerated fatty acid oxidation or inhibited fatty acid synthesis or both, as found for the STZ/HFD-32 fed mice [23]. 

### 3.3. VDAC1 Expression Levels in Langerhans Islets of STZ/HFD-32 Fed Mice 

**Figure 3:**
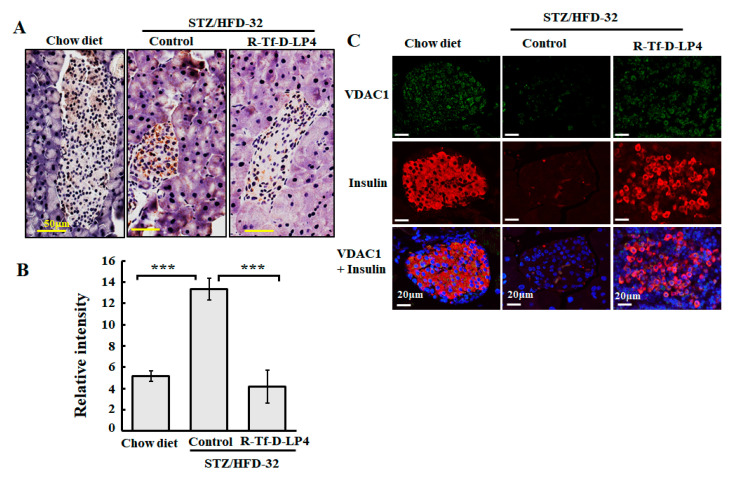
VDAC1 is overexpressed in Langerhans islets of STZ/HFD-32 fed mice. (**A**) Pancreatic sections from chow-fed, STZ/HFD-32 fed, and R-Tf-D-LP4 peptide-treated STZ/HFD-32 fed (14 mg/kg) mice were IHC stained for VDAC1 using specific antibodies followed by hematoxylin staining. (**B**) A quantitative analysis of VDAC1 staining intensity using a panoramic microscope and HistoQuant software (Quant Center 2.0 software, 3DHISTECH Ltd). Results are means ± SEM (n = 3–5; *** *p* ≤ 0.001). (**C**) Representative paraffin-embedded, formaldehyde-fixed pancreatic sections from chow diet-fed and STZ/HFD-32 fed mice with and without R-Tf-D-LP4 peptide treatment at the steatosis stage were IF co-stained with anti-VDAC1 and anti-insulin antibodies, and the nuclei were stained with DAPI.

We have previously demonstrated that the level of VDAC1 is upregulated in T2D organ donor islets and control islets under conditions of glucotoxicity, as well as in the T2D mouse model [42], and in livers of the STZ/HFD-32 fed mouse model [23]. Therefore, we examined the expression of VDAC1 in the pancreas of the STZ/HFD-32 diet-fed mice, representing the NAFLD mouse on a diabetic background and the effect of R-Tf-D-LP4 peptide treatment as compared to the levels in islets from chow diet-fed mice (Figure 3). Formaldehyde-fixed, paraffin-embedded pancreas sections were analyzed by IHC staining for VDAC1 using anti-VDAC1 specific antibodies. The results demonstrated increased staining in the islets of NAFLD mice relative to chow diet-fed mice (Figure 3A). However, this level decreased (Figure 3A) in mice treated with the R-Tf-D-LP4 peptide. In addition, the size of the islets was smaller in STZ/HFD-32 fed mice than in the chow diet-fed mice or in STZ/HFD-32 fed mice treated with R-Tf-D-LP4 (Figure 3A). A quantitative analysis of the staining intensity in the islets revealed a significant increase in VDAC1 staining in the STZ/HFD-32 fed mice as compared to both chow diet-fed and STZ/HFD-32 fed mice treated with the R-Tf-D-LP4 peptide (Figure 3B). 

Similar results were obtained using IF and co-staining for VDAC1 and insulin expression to better identify the insulin-producing islets. In this case, the results showed that the islets were disrupted and smaller in the STZ/HFD-32 fed mice than in the controls, but that mice treated with the R-Tf-D-LP4 peptide displayed a normal morphology and large islets (Figure 3C).

### 3.4. Effect of R-Tf-D-LP4 R-Tf-D-LP4 Peptide Treatment on Pancreatic Endocrine Activity in STZ/HFD-32fed Mice

**Figure 4:**
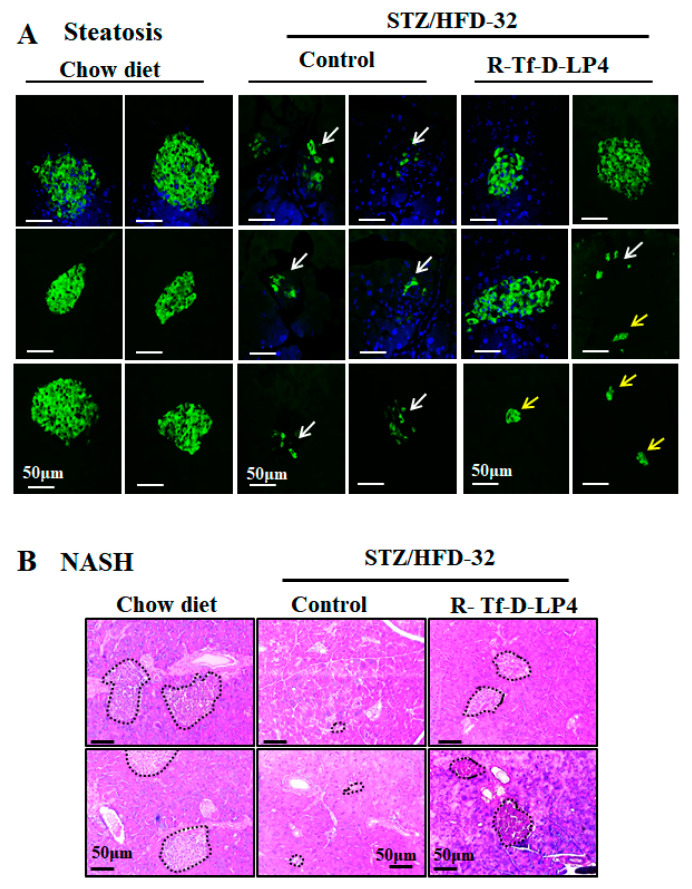
R-Tf-D-LP4 effect on the Langerhans islets in STZ/HFD-32 fed mice. (**A**) Representative paraffin-embedded, formaldehyde-fixed pancreas sections from chow-fed, or STZ/HFD-32 fed mice with and without R-Tf-D-LP4 peptide treatment. At the steatosis stage, sections were IF stained with anti-insulin antibodies, and the nuclei were stained with DAPI. White arrows point to disrupted islets, and yellow arrows point to small non-disrupted ones. (**B**) Pancreatic sections from chow-fed, STZ/HFD-32 fed mice with and without R-Tf-D-LP4 peptide treatment at the NASH stage were stained with H&E. These islets are circled.

In order to evaluate the endocrine activity of Langerhans islets in regulating blood glucose, we analyzed the size and numbers of these islets, as visualized by IF staining for insulin in the steatosis state (Figure 4A), and by H&E staining in NASH (Figure 4B) compared to chow diet-fed mice. As expected, there were large anti-insulin antibody stained clusters of islets in the pancreatic sections from chow-fed control animals (Figure 4). However, no such clusters were observed in the pancreases from STZ/HFD-32 fed mice at either the steatosis (Figure 4A) or NASH (Figure 4B) stage, and instead the islets appeared disrupted (Figure 4A, white arrows). After R-Tf-D-LP4 peptide treatment, both the size and number of Langerhans islets were increased. The results from several experiments with and without R-Tf-D-LP4 peptide treatment are summarized in Table 2. Langerhans islets were counted in each pancreas and categorized into several groups according to their size. Overall, the number of islets in pancreases of STZ/HFD-32 diet-fed mice in the steatosis or NASH stage was decreased by 50% and 40%, respectively, relative to the values in the chow diet-fed mice. R-Tf-D-LP4 peptide treatment increased the number of islets in mice with steatosis to the level of the chow-fed mice, and further increased the number by 30% in the NASH mice. An increase in both large and small islets was observed in the sections from R-Tf-D-LP4 peptide-treated mice, although most of this increase was in the small islets (Figure 4, yellow arrows and Table 2).

**Figure 5:**
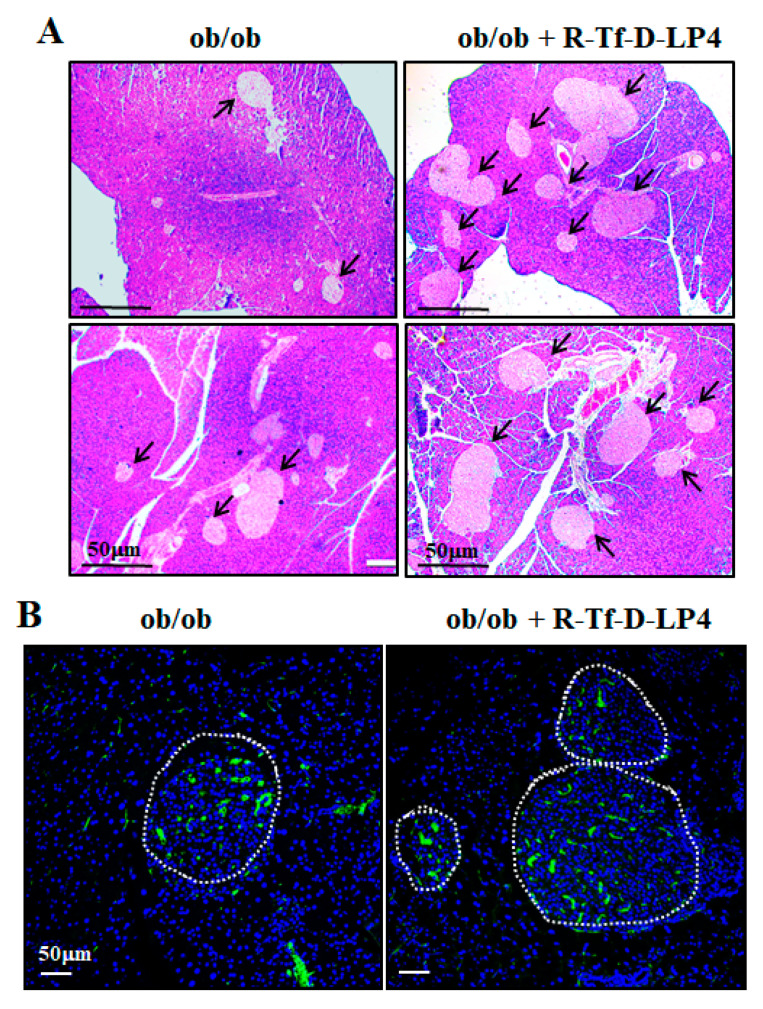
ob/ob mice show large islets where their number was increased by R-Tf-D-LP4 peptide treatment. ob/ob mice were treated or not treated with intravenous injections of the R-Tf-D-LP4 peptide (14 mg/kg) three times a week, mice were sacrificed, and the pancreases were removed and fixed. Representative paraffin-embedded, formaldehyde-fixed pancreatic sections were stained with Hematoxylin, eosin (**A**), or IF stained with anti-insulin antibodies, and the nuclei were stained with DAPI (**B**).

We also examined the effects of the R-Tf-D-LP4 peptide on the Langerhans islets of the ob/ob mice by hematoxylin, eosin (Figure 5A), and insulin (Figure 5B) staining of paraffin sections of the pancreas. As described previously [47,48], the size of the islets was significantly increased in the pancreas of ob/ob mice (Figure 5) compared to those of WT mice (Figure 5). R-Tf-D-LP4 peptide treatment of ob/ob mice increased the number of islets, as found for the STZ/HFD-32 R-Tf-D-LP4 peptide-treated mice (Figure 4, Table 2), and thereby also improved insulin production. This may explain the reduction in blood glucose induced by the peptide. 

**Figure 6:**
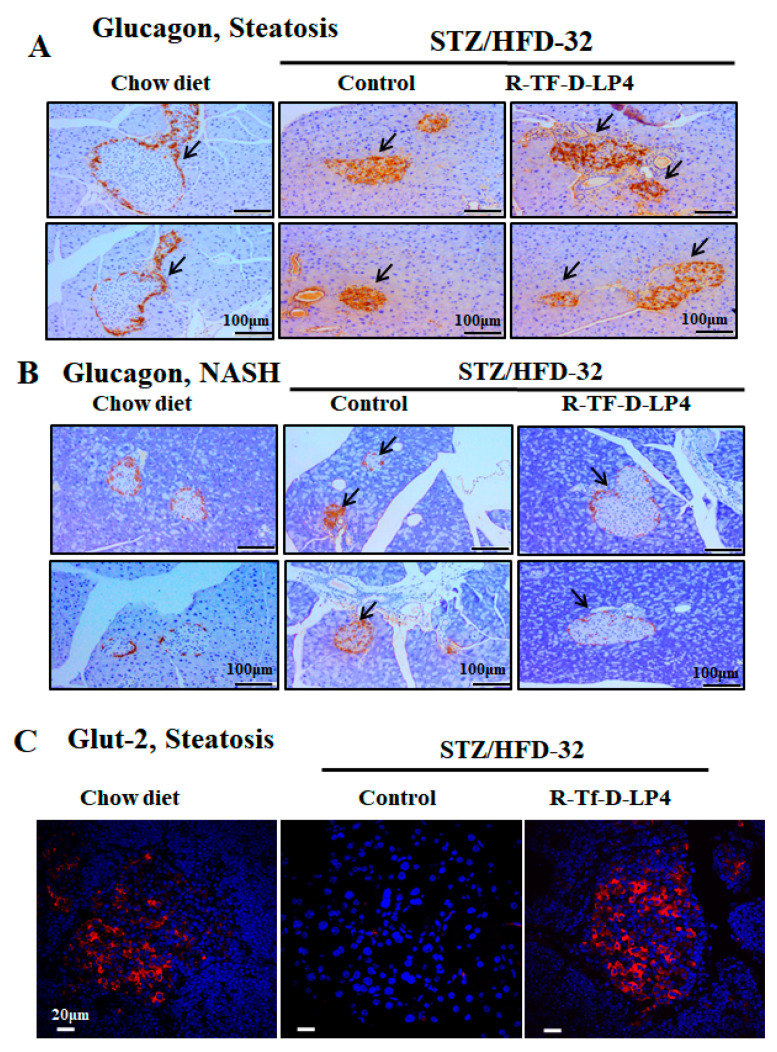
Glucagon and Glut-2 staining of Langerhans islets in STZ/HFD-32 fed mice at the steatosis or NASH stage and the effect of R-Tf-D-LP4 peptide treatment. Representative paraffin-embedded, formaldehyde-fixed pancreas sections from two mice from each of the experimental groups (chow-fed and STZ/HFD-32 fed mice with and without R-Tf-D-LP4 peptide treatment) at the steatosis (**A**) or NASH stage (**B**) were IHC stained with anti-glucagon antibodies. Arrows point to the islets. (**C**) Representative paraffin-embedded, formaldehyde-fixed pancreatic sections from chow-fed and STZ/HFD-32 fed mice with and without R-Tf-D-LP4 peptide treatment were IF stained with anti-Glut-2 antibodies, and the nuclei were stained with DAPI.

Normal islet of Langerhans morphology with the glucagon-producing α-cells present around the edge could be seen in glucagon IHC staining of the chow diet-fed mice (Figure 6A). However, in the STZ/HFD-32 fed mice with steatosis or NASH, the islets demonstrated an impaired morphology in which the glucagon-producing cells infiltrated into the islets (Figure 6A,B). This was improved to some extent in islets from the STZ/HFD-32 fed mice treated with R-Tf-D-LP4 at the steatosis stage (Figure 6A), but was completely restored in R-Tf-D-LP4 peptide-treated animals at the NASH stage, where the glucagon stained α-cells were localized as expected around the edge of the islet (Figure 6B).

Glut-2 encoded by SLC2A2 is predominantly expressed in hepatocytes, but also in kidney proximal convoluted tubule cells, intestinal absorptive cells, and pancreatic β-cells [49,50]. It is involved in glucose-sensing in pancreatic β-cells, the liver, and hypothalamus, as well as in triggering the glucose-mediated insulin secretion cascade [51]. This protein is thought to be involved in the pathogenesis of diabetes mellitus. Studies have reported that, in diabetic animal models, Glut-2 expression is down-regulated in pancreatic β-cells [52], while hepatic expression of this glucose transporter is enhanced [53]. In addition, mice lacking Glut-2 developed early diabetes and abnormal postnatal pancreatic islets [54], and loss of sugar detection by Glut-2 affects glucose homeostasis [55].

Here, we analyzed the expression of Glut-2 in the pancreas of STZ/HFD-32-fed mice with and without R-Tf-D-LP4 peptide treatment (Figure 6C). Glut-2 was present in the islets of the chow diet-fed mice, was not detected in the STZ/HFD-32 fed mice, but was re-expressed when the mice were treated with the R-Tf-D-LP4 peptide (Figure 6C). 

### 3.5. Effect of R-Tf-D-LP4 Treatment on Proliferation of Islet Cells 

**Figure 7:**
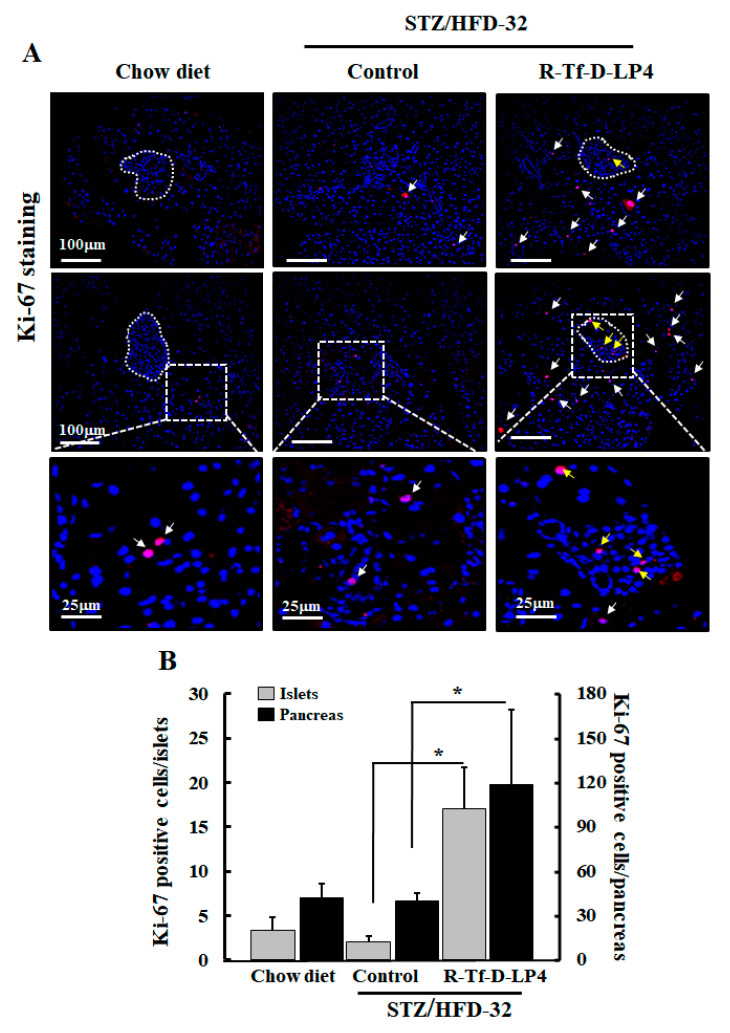
Effect of R-Tf-D-LP4 on the expression levels of the proliferation marker Ki-67 in the pancreas of STZ/HFD 32 fed mice. (**A**) Sections from two mice from each of the experimental groups (chow-fed and STZ/HFD-32 fed mice with and with R-Tf-D-LP4 peptide treatment) at the steatosis stage were IF stained with anti-Ki-67 antibodies and with DAPI for nuclear staining. The islets are circled, and white and yellow arrows point to Ki-67 staining of cells located around the periphery or inside the Langerhans islets, respectively. (**B**) Ki-67 positive cells inside and around the edge of the Langerhans islets were counted in the whole sections. Results are means ± SEM (n= 3–5 pancreases; * *p* ≤ 0.05).

One of the possible explanations for the increase in islet size and number is the proliferation/regeneration of β-cells or other cell types composing the Langerhans islets such as α-cells. A known marker for cell proliferation is the Ki-67 protein. Accordingly, to evaluate proliferation, pancreatic sections were IF stained using specific anti-Ki-67 antibodies. Counting the Ki-67 positive cells in the pancreatic sections from chow diet-fed mice and STZ/HFD-32 fed mice, revealed similarly low numbers and distribution of the Ki-67 positive cells inside and in the periphery of the islets (Figure 7A, white arrows). However, R-Tf-D-LP4-treated mice showed that Ki-67 positive cells increased both in the islet interior and also in cells outside of the islets (Figure 7A, white arrows) by about 8-fold and 3-fold, respectively, compared to both chow diet-fed mice and STZ/HFD-32 fed ones (Figure 7B). 

### 3.6. Effect of R-Tf-D-LP4 Peptide Treatment on β- and α-cell Development

**Figure 8:**
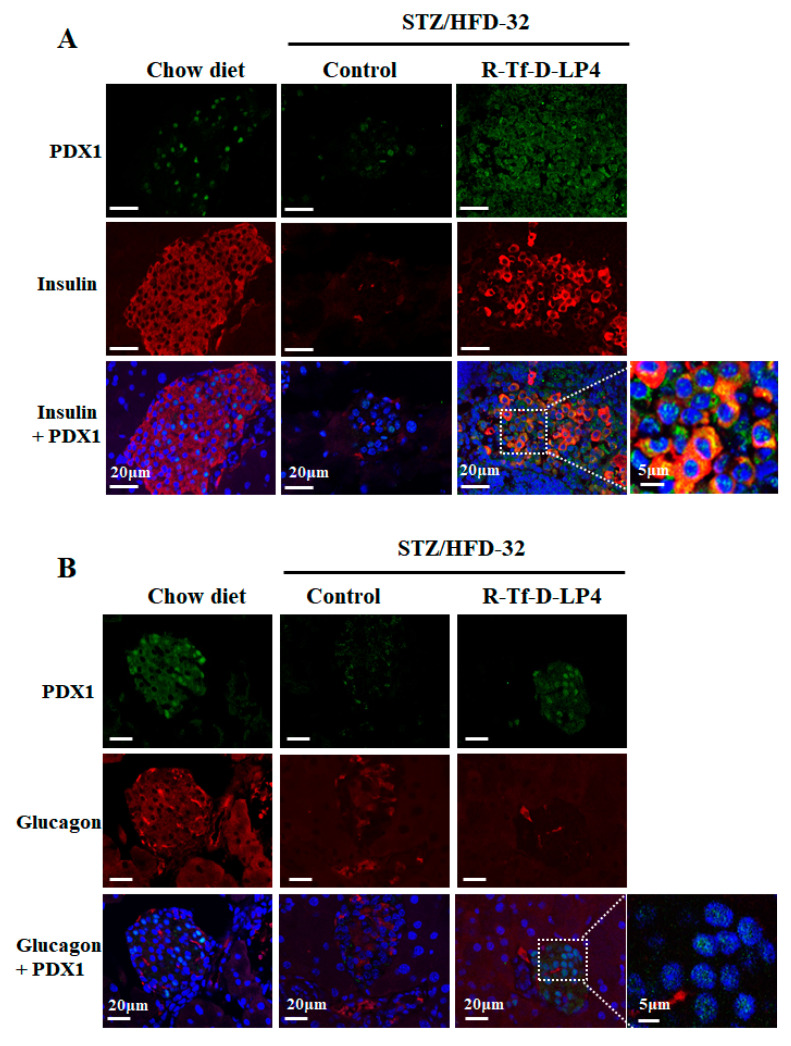
PDX1 expression is increased in islets of Langerhans by R-Tf-D-LP4 peptide treatment. (**A**) Representative pancreatic sections from 3–5 mice from each experimental group (chow-fed, STZ/HFD-32 fed, and STZ/HFD-32 fed treated with the R-Tf-D-LP4 peptide) were IF stained with anti-PDX1 (green), and anti-insulin (red) antibodies and the nuclei were stained with DAPI. (**B**) A similar experiment with sections IF stained with anti-PDX1 (green), and anti-glucagon (red) antibodies, and the nuclei stained with DAPI. Enlargement of selected image areas are presented to clearly demonstrate that PDX1 was also localized in the nucleus.

Another possible explanation for the increase in the number and size of insulin-producing cells in the Langerhans islets following R-Tf-D-LP4 peptide treatment is the differentiation of precursor cells or the trans-differentiation of differentiated cells such as α-cells into insulin-producing β-cells. One of the markers of β-cell maturation and necessary for pancreatic development is PDX1 (pancreatic and duodenal homeobox 1), also known as insulin promoter factor 1. This is a transcription factor that enhances the expression of the insulin-encoding gene (INS) among other functions [56]. In order to assess the impact of R-Tf-D-LP4 peptide treatment on PDX1 expression in β- and α-cells, pancreatic sections were co-stained for PDX1 and insulin (Figure 8A), or glucagon (Figure 8B), using specific antibodies. In contrast to the high level of PDX1 staining in the pancreases from chow diet-fed mice, PDX1 was strongly decreased in STZ/HFD-32 fed mice (Figure 8A). However, pancreases obtained from STZ/HFD-32 fed mice treated with the R-Tf-D-LP4 peptide showed a higher level of staining for PDX1, with some but not all of the cells co-stained for insulin (Figure 8A).

Glucagon staining in chow diet-fed mice showed the expected staining in the islet periphery, but the staining tended to be in the islet interior in STZ/HFD-32 fed mice, with and without R-Tf-D-LP4 peptide treatment (Figure 8B), as shown above for glucagon staining (Figure 6A). As already discussed, PDX1 staining was high in chow diet-fed mice, strongly decreased in STZ/HFD-32 fed mice, and highly increased in mice treated with the R-Tf-D-LP4 peptide over the level in the chow-fed mice (Figure 8B). These results suggest that R-Tf-D-LP4 peptide treatment increases the expression of the transcription factor PDX1, thereby enhancing the expression of the insulin-encoding gene. 

## 4. Discussion

NAFLD is primarily associated with obesity, diabetes, and metabolic syndrome [57,58]. In our previous study, we demonstrated the effectiveness of the VDAC1-based peptides, Tf-D-LP4, in treating steatosis and NASH as induced in STZ/HFD-32 fed mice [23]. The tight connection between NAFLD, a major factor in the pathogenesis of T2D, and mitochondria was well demonstrated in that study [23]. We showed that treatment of STZ/HFD-32-fed mice with the R-Tf-D-LP4 peptide affected carbohydrate and lipid metabolism, and increased the expression of enzymes and factors associated with mitochondrial fatty acid transport, enhancing β-oxidation and thermogenic processes. These peptide effects involving mitochondria are in agreement with the finding that the mitochondrial protonophore 2,4 dinitrophenol (DNP) can reverse diabetes and steatohepatitis in pre-clinical models, but it is too toxic for clinical use [59].

Here, we focused on the effects of the R-Tf-D-LP4 peptide on restoring blood glucose levels to close to normal, and improved the state of the Langerhans islets with respect to size, number, and insulin production. These effects of the R-Tf-D-LP4 peptide could be due to general elimination of the steatosis/NASH pathologies in the liver, reducing fat accumulation in hepatic cells, lowering inflammation, reducing gluconeogenesis, and increasing glycogen synthesis [23]. However, the results with insulin-stained islets suggest that R-Tf-D-LP4 peptide treatment of the STZ/HFD-32 fed mice induces β-cell production (Figure 4, Table 2). 

The islets of Langerhans [60] comprise glucagon-producing α-cells located in the periphery of the islet, insulin-producing β-cells in the interior, and somatostatin-producing γ-cells that are evenly distributed across the islet [61]. 

The increase in the size of the islets and budding of new islets by R-Tf-D-LP4 peptide treatment of STZ/HFD-32 fed mice raised the question as to the source of these β-cells. The possibility that they might regenerate is controversial and is currently a subject of debate [62,63]. One leading theory suggests that the formation of new islets is caused by the differentiation of progenitor or stem cells to β-cells [64], while an opposing theory suggests that differentiated cells in the pancreas or liver undergo a process of trans-differentiation by which cells de-differentiate and then re-differentiate to β-cells [63,65]. Another possible explanation is proliferation of β-cells, although the total β-cell proliferation rate was observed to be low in both mice and humans, as only a fraction of these cells proliferate [66]. In order to investigate the source of the observed increased number and size of the β-cell containing islets after R-Tf-D-LP4 peptide treatment of STZ/HFD-32 fed mice, we examined the expression of insulin and glucagon (Figure 4, Figure 6 and Table 2), as well as the proliferation factor Ki-67 (Figure 4, Figure 6, Figure 7), the transcription factor regulator of normal pancreatic development, and the β-cell differentiation marker, PDX1, activator of a number of genes, including insulin, somatostatin, glucokinase, and glucose transporter type 2 (Glut-2) [67] (Figure 8). The results demonstrated high numbers of Ki-67 positive cells both inside and outside of the islets from R-Tf-D-LP4-peptide-treated STZ/HFD-32 fed mice (Figure 7). This implies that the observed increase in β-cell content in the islets of these mice is due not only to increased proliferation of cells within the islets, but also of other cells outside of the islets that may explain the increase in the number of small islets (Figure 4, Table 2). 

The human diabetes gene and homeodomain protein, PDX1, has a well-described role in the function and survival of β-cells, where it is a key regulator of normal pancreatic development and β-cell differentiation, inducing differentiation of both embryonic stem cells and bone marrow-derived mesenchymal stem cells into insulin-producing cells [63,65,68]. PDX1 is transiently expressed during the development of the pancreas and duodenum, and in the differentiation and maturation of β-cells, and acts as an enhancer for several genes including the insulin-transcribing gene [69]. Thus, in the adult pancreas, it is responsible for the regulation of genes that are essential for islet development, function, proliferation, and maintenance of glucose homeostasis [70,71]. Accordingly, downregulating PDX1 expression in humans and in animal models results in T2D, β-cell dysfunction, and impaired islet compensation in the presence of insulin resistance [72].

The role of PDX1 in β-cell survival in response to HFD has already been established [73]. While PDX1 expression itself is not induced by stress, it regulates islet compensation for HFD-induced insulin resistance, in part through direct transcriptional regulation of Atf4 and Wsf1 [73]. Here, co-staining the pancreas of STZ/HFD-32 fed mice, with anti-PDX1 and anti-insulin, or anti-PDX1 and anti-glucagon antibodies revealed a significant increase of PDX1 expression in the STZ/HFD-32 diet-fed mice treated with the R-Tf-D-LP4 peptide (Figure 8). PDX1 expression was mainly in the β-cells, suggesting that the source of the increased number of these cells upon R-Tf-D-LP4 peptide treatment is most likely due to their proliferation. However, Ki-67-expressing cells were increased upon R-Tf-D-LP4 peptide treatment of STZ/HPD-32 fed mice not only in the islets, but also in cells outside of them (Figure 7). This may suggest that other cell types undergo differentiation into β-cells, resulting in the increase of new small islets (Figure 4 and Table 2). Identification of the precursor of these β-cells will require additional study. 

**Figure 9:**
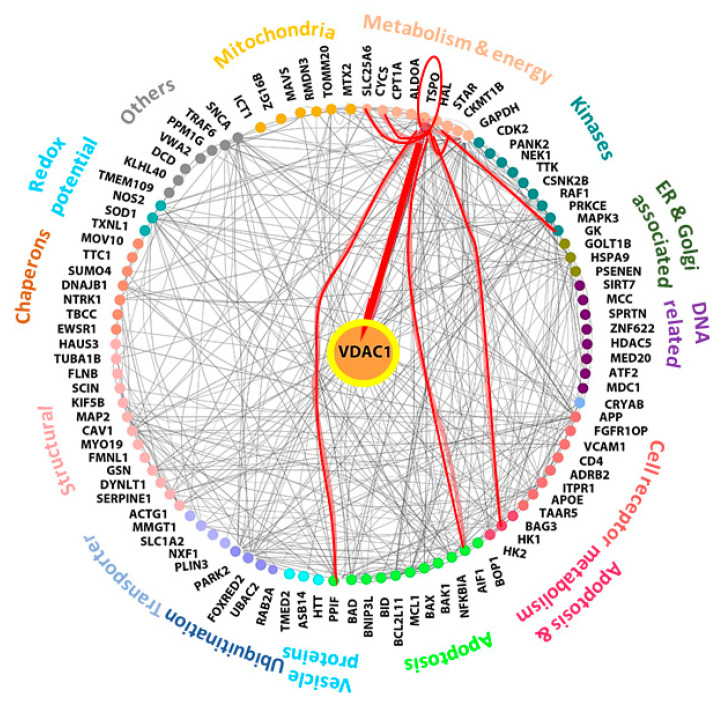
Network analysis of VDAC1-interacting proteins and TSPO. Networks of proteins shown to interact with VDAC1 were produced using https://cytoscape.org/. Experimentally determined protein–protein interactions are represented by lines. Proteins sharing similar or related functions are grouped and similarly colored and the group-associated function is indicated as proteins involved in metabolism, apoptosis, signal transduction, cell receptors, transport, chaperons, and kinases among others. The red lines connect TSPO to its protein interactions as predicted by curated databases. TSPO-connected proteins include: AIF1 (Apoptosis-inducing factor 1), HK-I (hexokinase-1), GK (glycerol kinase), StAR (steroidogenic acute regulatory proteins), VDAC1 (voltage-dependent anion channel 1). PPIF (peptidyl-prolyl cis-trans isomerase, cyclophilin D), CyCs (cytochrome c), and SLC25A6 (ANT, ATP/ADP translocase).

Considering the question of how the VDAC1-based peptide R-Tf-D-LP4 induces the proliferation of β-cells or induces differentiation of other cell types to β-cells, we assume that this relates to the ability of VDAC1 to interact with over 100 protein partners [4,5]. VDAC1 functions as an anchoring site for diverse sets of cytosolic, ER, and mitochondrial proteins that together mediate and/or regulate metabolic, apoptotic, and other processes in normal and diseased cells. The VDAC1 interactome includes proteins involved in metabolism, apoptosis, signal transduction, and anti-oxidation, as well as DNA- and RNA-associated proteins among others [4,5] (Figure 9). Thus, the R-Tf-D-LP4 peptide may compete with VDAC1 for one or more of these proteins, and thus modulate their function by changing or preventing their interaction with VDAC1. In this context, we have previously demonstrated the interaction of VDAC1 with long-chain acyl-CoA synthetase (ACSL) and carnitine palmitoyltransferase 1a (CPT1a) proteins in the STZ/HFD-32 fed mouse model [23]. The VDAC1-interacting proteins involved in β-cell regeneration and islet number and size restoration leading to normalization of blood glucose levels are yet to be identified.

One of the proteins that interacts with VDAC1 and with another seven VDAC1-interacting proteins is the translocator protein (TSPO), previously known as the peripheral benzodiazepine receptor (Figure 9). The cytoscape database indicates that TSPO interacts with a number of proteins associated with metabolism and energy homeostasis including HK-I (hexokinase-1), GK (glycerol kinase), and ATP/ADP translocase (ANT) [74]. It also interacts with or mediates the action of StAR [75]. In addition, TSPO interacts with apoptosis-regulating proteins including cytochrome c (CyCs), peptidyl-prolyl cis-trans isomerase, cyclophilin D (PPIF), and apoptosis-inducing factor 1 (AIF). Except for VDAC1, the interactions of TSPO with the other proteins are predicated and not based on measurements of direct interactions. 

TSPO also has been implicated in the diabetes-inducing neurophysiological and structural changes in the central nervous system (CNS) [76], and is associated with cognitive deficits and increased risk of dementia, stroke, cerebrovascular events, Alzheimer’s disease, and psychiatric disorders [77,78,79]. In a study of diabetics, TSPO activation was effective in ameliorating the disease severity through a local increase in neuroactive steroids [80]. It was postulated that TSPO in adipose tissues could serve as a pharmacological target in the treatment of T2D [81]. This is supported by the observations that TSPO ligands were shown to display neuroprotective properties in models of toxic and diabetic-induced neuropathies [82]. These ligands were shown to improve glucose uptake and adipogenesis through TSPO activation and to display neuroprotective properties in models of toxic and diabetic-induced neuropathies [82]. The anti-diabetic effects of the ligands was shown to be mediated via modulation of mitochondrial function, and in particular, cholesterol transport, thereby improving the biogenesis of the lipid bilayer [81].

In a similar model used in this study, the STZ/HFD in rats, the role of TSPO in the treatment of depression in T2D was evaluated by demonstrating the pharmacological effects of AC-5216, a ligand for TSPO. The anxiolytic- and antidepressant-like effects produced in animal models were antagonized by PK11195 (a TSPO antagonist) [83].

To conclude, we were able to demonstrate that in a NAFLD mouse model produced by STZ/HFD-32—and thus having a diabetic background—treatment with the R-Tf-D-LP4 peptide reduced blood glucose levels, increased the insulin content of islets, improved their quantity and size, and restored their morphology. In addition, the increase of the cell proliferation marker, Ki-67 positive cells in the pancreas of R-Tf-D-LP4 peptide-treated mice suggests that the R-Tf-D-LP4 peptide may promote the formation of new β-cells. Moreover, the R-Tf-D-LP4 peptide-induced expression of PDX1 may regulate the expression of genes essential for islet development, function, proliferation, and maintenance of glucose homeostasis [70,71]. Further studies are required in order to explore the mechanism by which this is achieved. 

The main therapeutic goal for diabetes drugs is to reduce blood glucose levels in patients. Although it is important to elucidate the mechanisms that underline the increase in β-cells induced by the R-Tf-D-LP4 peptide treatment in this STZ/HFD-32 fed model, the current reduction of blood glucose levels means that the R-Tf-D-LP4 peptide may represent a potential treatment for diabetes. As a next step, it would be quite interesting to test the effects of the R-Tf-D-LP4 peptide using other mouse models of diabetes. Moreover, a comparison of the proteins differentially expressed in Langerhans islets from control and HFD-32 mice with and without R-Tf-D-LP4 peptide treatment is expected to provide further insight into the signaling pathways modified by this treatment. 

## Figures and Tables

**Table 1:** All antibodies, their source, and dilution used in this research.

Antibody	Source and Catalog No.	Dilution IHC, IF
Mouse anti-Glucagon	Abcam, Cambridge, UK, ab10988	1:500
Mouse anti-Insulin	Abcam, Cambridge, UK, ab6995	1:300
Rabbit anti-Ki-67	Abcam, Cambridge, UK, ab15580	1:250
Rabbit anti-PDX1	Abcam, Cambridge, UK, ab47267	1:300
Rabbit anti-VDAC1	Abcam, Cambridge, UK, ab15895	1:500
Donkey anti-Mouse Alexa Fluor 488	Abcam, Cambridge, UK, ab150109	1:500
Goat anti-Rabbit Alexa Fluor 555	Abcam, Cambridge, UK, ab150086	1:500
Donkey anti-Mouse HRP conjugated	Abcam, Cambridge, UK, ab98799	1:500
Goat anti-Rabbit HRP conjugated	Promega, Madison, WI, W401B	1:500
Mouse anti-transporter GLUT2	Abcam, Cambridge, UK, ab85715	1:500

**Table 2:** Effect of R-Tf-D-LP4 peptide treatment on the number and size of Langerhans islets in STZ/HFD-32 diet-fed mice with steatosis or NASH. Insulin-stained Langerhans islets were counted, measured, and categorized according to their size. The number of total islets counted per pancreas is presented. ns indicates not-significant.

	**Steatosis**		
**Parameter**	**Chow**	**HFD-32**	**HFD-32, Peptide**
Total no. of islets	136 (n = 5)	217 (n = 16)	451 (n = 17)
Average no. of islets per mouse	27	14 (<0.001)	27 (ns)
Islet size, 1–50 mm	11	4 (<0.05)	16 (ns)
Islet size, 51–100 mm	8	6 (<0.05)	8 (ns)
Islet size, 101–150 mm	5 (ns)	3 (ns)	2 (ns)
Islet size, 151–300 mm	3	1 (<0.05)	1 (ns)
	**NASH**		
**Parameter**	**Chow**	**HFD-32**	**HFD-32, peptide**
Total no. of islets	136 (n = 5)	168 (n = 10)	354 (n = 10)
Average no. of islets per mouse	27	17 (<0.001)	35 (<0.05)
Islet size, 1–50 μm	11	8 (ns)	16 (ns)
Islet size, 51–100 μm	8	6 (ns)	12 (ns)
Islet size, 101–150 μm	5	2 (ns)	4 (ns)
Islet size, 151–350 μm	3	1 (ns)	3 (ns)
