# Correction: Pittala et al. The VDAC1-based R-Tf-D-LP4 Peptide as a Potential Treatment for Diabetes Mellitus. *Cells* 2020, *9*, 481

**DOI:** 10.3390/cells13191630

**Published:** 2024-09-30

**Authors:** Srinivas Pittala, Idan Levy, Soumasree De, Swaroop Kumar Pandey, Nataly Melnikov, Tehila Hyman, Varda Shoshan-Barmatz

**Affiliations:** Department of Life Sciences and the National Institute for Biotechnology in the Negev, Ben-Gurion University of the Negev, Beer-Sheva 84105, Israel; sirinivas9@gmail.com (S.P.); idan0@post.bgu.ac.il (I.L.); soumasree.de@gmail.com (S.D.); pandey@post.bgu.ac.il (S.K.P.); chepovetsky93@gmail.com (N.M.); tehilahm@post.bgu.ac.il (T.H.)

## Error in Figure

In the original publication [1], the image in Figure 1D is overlapping with Figure 1E [2]. The images have been inadvertently overleaped with previously published work. The corrected Figure 1D, R-Tf-D-LP4 has now been replaced with a different image, shown below. 

The authors state that the scientific conclusions are unaffected. This correction was approved by the Academic Editor. The original publication has also been updated.

## Figures and Tables

**Figure 1:**
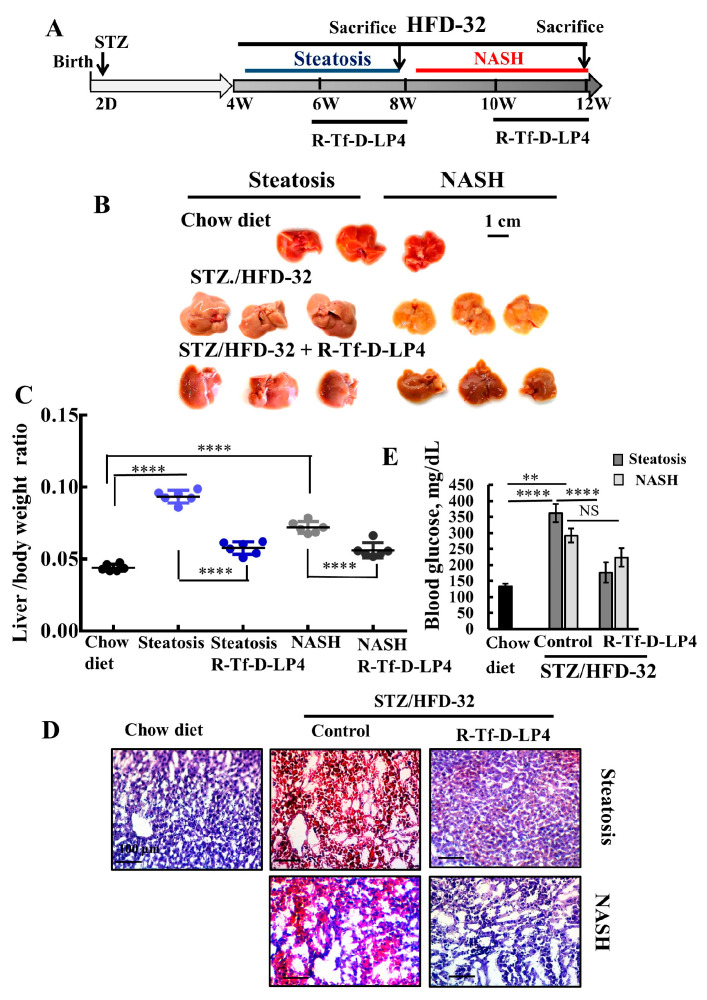
R-Tf-D-LP4 peptide-mediated inhibition of steatotic and non-alcoholic steatohepatitis (NASH) liver pathology in a STZ/HFD-32 mouse model. (**A**). Schematic presentation of the course of steatosis and NASH induced by a STZ/HFD-32 diet and the effect of R-Tf-D-LP4 peptide treatment. (**B**–**D**). Liver from mice fed with chow (normal diet), HFD-32, or HFD-32 and treated with the R-Tf-D-LP4 peptide (14 mg/kg) by i.v. injection every two days from Week 6 to 8 for steatosis and from Week 8 to 10 for NASH, as described in the Methods section. Mice were then sacrificed, livers were removed, photographed (**B**)**,** and weighed (**C**) Results are means ± SEM (n = 10), (*p* **** ≤ 0.0001). Representative liver sections were stained with Oil Red O (**D**). Blood glucose level of mice was measured. Results are means ± SEM (n = 5–10; ** *p* ≤ 0.01, *p* **** ≤ 0.0001) (**E**).
